# Long-term age-dependent behavioral changes following a single episode of fetal N-methyl-D-Aspartate (NMDA) receptor blockade

**DOI:** 10.1186/1471-2210-4-28

**Published:** 2004-10-28

**Authors:** G Andrew Mickley, Cynthia L Kenmuir, Colleen A McMullen, Alicia Snyder, Anna M Yocom, Deborah Likins-Fowler, Elizabeth L Valentine, Bettina Weber, Jaclyn M Biada

**Affiliations:** 1 Department of Psychology and the Neuroscience Program, Baldwin-Wallace College, 275 Eastland Road, Berea, OH 44017-2088, USA

## Abstract

**Background:**

Administration of the N-methyl-D-aspartate (NMDA) antagonist ketamine during the perinatal period can produce a variety of behavioral and neuroanatomical changes. Our laboratory has reported reliable changes in learning and memory following a single dose of ketamine administered late in gestation. However, the nature of the drug-induced changes depends on the point during embryonic development when ketamine is administered. Embryonic day 18 (E18) rat fetuses pre-treated with ketamine (100 mg/kg, i.p. through the maternal circulation) and taught a conditioned taste aversion (CTA) learn and remember the CTA, whereas E19 fetuses do not. The current study sought to determine if long-term behavioral effects could be detected in animals that received ketamine or a saline control injection on either E18 or E19. Rat behavior was evaluated on two different measures: spontaneous locomotion and water maze learning. Measurements were collected during 2 periods: *Juvenile *test period [pre-pubertal locomotor test: Postnatal Day 11 (P11); pre-pubertal water maze test: P18] or *Young-adult *test period [post-pubertal locomotor test: P60; post-pubertal water maze test: P81].

**Results:**

Water maze performance of ketamine-treated rats was similar to that of controls when tested on P18. Likewise, the age of the animal at the time of ketamine/saline treatment did not influence learning of the maze. However, the young-adult water maze test (P81) revealed reliable benefits of prenatal ketamine exposure – especially during the initial re-training trial. On the first trial of the young adult test, rats treated with ketamine on E18 reached the hidden platform faster than any other group – including rats treated with ketamine on E19. Swim speeds of experimental and control rats were not significantly different. Spontaneous horizontal locomotion measured during juvenile testing indicated that ketamine-treated rats were less active than controls. However, later in development, rats treated with ketamine on E18 were more active than rats that received the drug on E19.

**Conclusion:**

These data suggest that both the day in fetal development when ketamine is administered and the timing of post-natal behavioral testing interact to influence behavioral outcomes. The data also indicate that the paradoxical age-dependent effects of early ketamine treatment on learning, previously described in fetuses and neonates, may also be detected later in young adult rats.

## Background

Administration of ketamine or other NMDA receptor blocking drugs [1] may bring with it both beneficial and problematic outcomes. Ketamine's use as a dissociative anesthetic is well established in clinical practice [1] and more recently, it has also been proposed as a neuroprotectant against hypoxic-ischemic brain damage in neonatal rats [2]. However, in adult animals, NMDA receptor blockade is known to produce psychotomimetic side effects [3], impair memory formation [4-7], and may produce neurotoxicity [3,8-11]. This neurotoxicity is evidenced by vacuolization of cortical neurons [3,10] and has also been linked to programmed cell death (apoptosis) during development [12-14].

The toxic effects of NMDA receptor blockade are apparently dependent on the dose of the drug, administration regimen, and the age of the animal treated. For example, vulnerability to MK-801-induced cortical vacuolization is not evident in fetal animals but rather begins at approximately the time of puberty [8]. On the other hand, apoptogenic effects of ketamine have been seen following drug administrations during the last trimester of pregnancy [12]. Further, the selection of an acute or chronic dosing regimen may also modulate the neurobehavioral outcomes and the permanence of the neurological changes that can be expected [9,13,15-18].

Recent experiments from our laboratory have focused on age-dependent effects of a single dose of ketamine on fetal learning and memory. Rat fetuses can learn conditioned taste aversions (CTAs) and exhibit taste-mediated conditioned motor responses (CMRs) [19,20], which can be modulated in complex ways by exposure to ketamine at different times during the perinatal period [20,21]. For example, ketamine will either cause a potentiation or a blockade of memory formation in rats, depending on the specific day during fetal development when the drug is administered. Rat fetuses that receive ketamine on E18 (30-minutes before CS-US pairing) are able to learn and remember CTAs and CMRs quite well. However, rat fetuses that receive ketamine before CTA training just one day later, on E19, exhibit an amnesia for these conditioned responses [21,22]. We have referred to this phenomenon as the "ketamine paradox" [21].

These previous studies have only tested the durability of the ketamine paradox over a period of up to 2 weeks [22] and have looked at a very narrow range of behavioral measures – all with gustatory components. However, there are some indications that early treatment with NMDA receptor blocking drugs can have long-term behavioral implications. For example, neonatal treatment with MK-801 can produce long-lasting behavioral radioprotection in rats with x-ray-induced hippocampal damage [23]. Likewise, Maier et al. [24] have reported that MK801-induced NMDA receptor antagonism in young rats (P7-17) extends the sparing of hindlimb function after spinal transection in older animals. However, treatment with an NMDA receptor antagonist [(+)HA-966] for a longer time, later in neonatal development (P10-20), impaired motor and cognitive behaviors in adult rats [25]. Several questions remain. What is the range of behaviors that can be influenced by ketamine treatment during the perinatal period? How long lasting are the different behavioral effects of ketamine administered late in gestation?

The current study extends our original observations and reports how a single injection of ketamine on either E18 or E19 modulates spontaneous locomotor activity and performance in a water maze. We tested the rats as juveniles and then as young adults. Our data suggest several age-dependent effects of early ketamine treatment – effects that depend on not only the length of time between drug administration and behavioral testing but also on the day in embryonic development when the NMDA receptor antagonist was administered.

## Results

### Water maze

#### P18 water maze test

**Figure 1:**
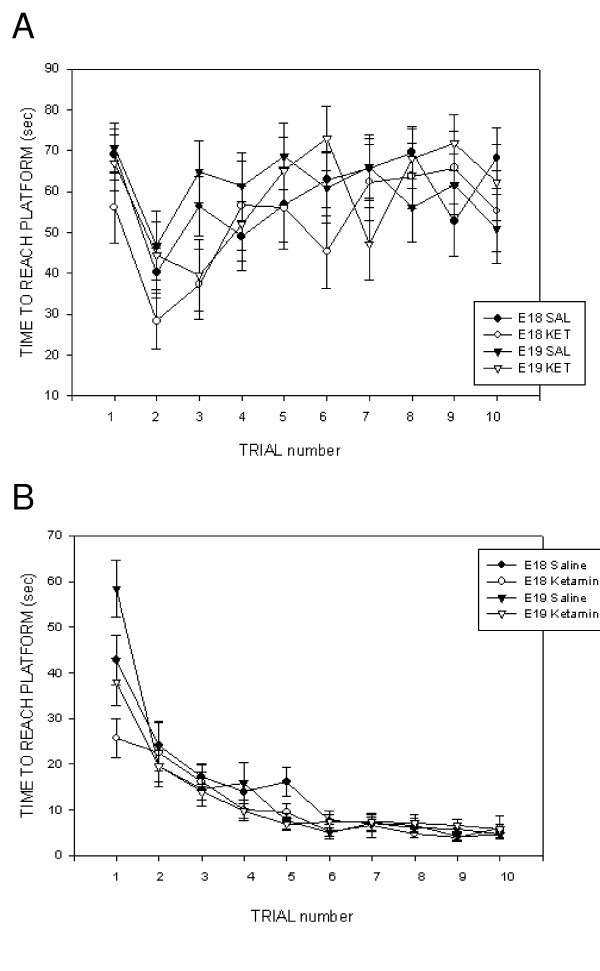
**Time to mount a hidden platform during the first (juvenile; panel A) and second (young-adult; panel B) water maze test. **Rats were treated (through the maternal circulation) with either 100 mg/kg ketamine HCl (i.p.) or saline on either E18 or E19 and then tested later on P18 and P81. The data presented here illustrate raw latencies without taking into account the time the animals spent treading water (i.e., not making forward progress). **Panel A: **P18 rats generally decreased their latencies to mount the hidden platform over the 10 trials. This effect was most prominent over the first 3 trials. The behavioral changes induced by drug or age manipulations were not statistically significant. **Panel B: **P81 rats all readily re-learned the location of the hidden platform as there was a significant reduction in time to mount the platform over the 10 trials. The analysis (see text) also revealed a significant Age X Drug interaction which was most prominent on trial 1 (see also Figure 3). Data were analyzed using a three-way ANOVA [Drug (100 mg/kg ketamine HCl, saline control) X Treatment age (E18, E19) X Time] with time blocks as the repeated time factor and compensation for unequal Ns. Variance indicators are the Standard Error of the Mean (SEM).

**Figure 2:**
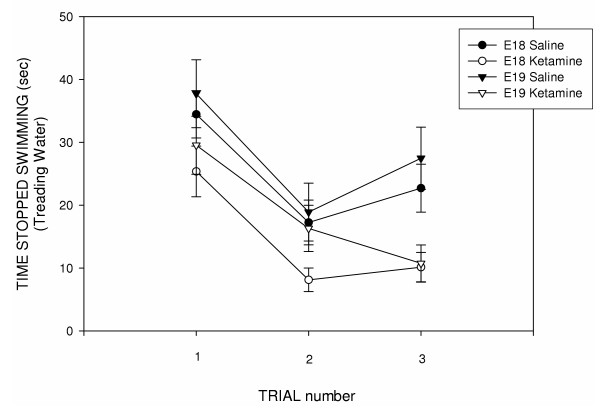
**Time spent treading water (i.e., not making forward progress) during the P18 water maze test. **There was a general decline in the time spent treading water as the animals learned the maze. Ketamine-treated rats spent the least time exhibiting this behavior. Rats treated with the NMDA-receptor blocking drug on E18 stopped swimming for the shortest period and made more-constant progress towards the hidden platform. Data were analyzed using a three-way ANOVA [Drug (100 mg/kg ketamine HCl, saline control) X Treatment age (E18, E19) X Time] with time blocks as the repeated time factor and compensation for unequal Ns. Variance indicators are the Standard Error of the Mean (SEM).

Over the10 trials of the water maze test, subjects significantly reduced their latencies to mount the hidden platform [F(9, 102) = 4.233; p < 0.0001] indicating the gradual learning of the maze. However, as Figure 1A illustrates, the animals never gained real proficiency on this task. The latencies decreased most dramatically within the first 3 trials and therefore we undertook a more in-depth analysis of this portion of the study. We also noticed that, as the animals swam, they would sometimes stop and tread water against the side of the tank. Therefore, we undertook an analysis of the stop time/trial during the first 3 water maze trials (see Figure 2). The time spent treading water decreased significantly over the first 3 trials [F(2, 111) = 18.359; p < 0.001]. There was also a significant drug effect indicating that ketamine-treated rats spent less time treading water than did saline-treated controls [F(1, 153) = 15.972; p < 0.001]. This effect was independent of the age at which the rats received ketamine.

Time spent treading water at the side of the tank may be interpreted as an alternative, futile, escape strategy and not necessarily as an indicator of learning the position of the hidden platform. Subsequent analyses subtracted out stop-times in order to provide the most accurate portrayal of maze learning during the first 3 trials. With stop-time removed, the declining time-to-platform [F(2, 102) = 3.501; p = 0.034] and swimming distance [F(2, 102) = 4.632; p = 0.012] over the first 3 trials indicated a learning of the maze during this initial exposure to the apparatus. However the behavioral changes induced by drug or age manipulations were not statistically significant. The number of trials in which the rat failed to mount the platform within 90 seconds was not significantly different among the 4 treatment groups. Likewise, swim speed did not decrease significantly over the first 3 maze trials indicating that the animals were not fatiguing as they undertook multiple swims.

Once the rat mounted the hidden platform we timed how long the subject remained there before it was removed (maximum of 30 seconds). An analysis of these data during the first 3 maze trials revealed neither a significant influence of subject age nor drug treatment.

#### P81 water maze test

**Figure 3:**
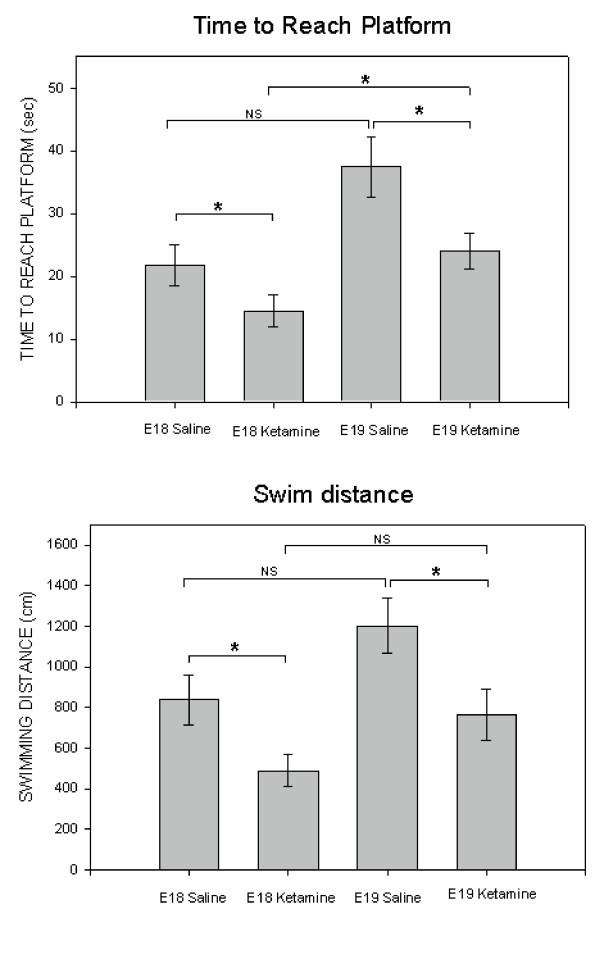
**Time to mount the hidden platform (top panel) and swimming distance to the platform (bottom panel) during the first trial of the second (young-adult) water maze test. **Rats were treated (through the maternal circulation) with either 100 mg/kg ketamine HCl (i.p.) or saline on either E18 or E19 and then tested later on P81. The temporal data presented here do not include time spent treading water but rather represent only the time that the rats were making forward progress. Ketamine-treated rats found the platform significantly faster than saline controls and swam shorter distances to do so. Group comparisons indicated that rats treated with ketamine on E18 or E19 reached the hidden platform significantly faster and swam shorter distances than saline-treated controls (* = p ≤ 0.05; NS = non-significant group differences). Further, E18 rats treated with ketamine later exhibited a shorter latency to reach the hidden platform than did E19 rats treated with ketamine. Data were analyzed using a two-way ANOVA [Drug (100 mg/kg ketamine HCl, saline control) X Treatment age (E18, E19)]. Individual group comparisons were accomplished by using t-tests employing the Bonferroni compensation for multiple comparisons. Variance indicators are the Standard Error of the Mean (SEM).

An analysis of the second water maze test indicated that there was a significant reduction in time [F(9, 157) = 26.868; p < 0.001] to mount the hidden platform over the 10 trials (see Figure 1B). The analysis also revealed a significant Age X Drug interaction [F(1, 401) = 5.15; p = 0.024] but no significant main effects of drug or age at treatment. Rats had previous experience with the maze (see P18 maze data) and inspection of the data indicated that, after the first trial, latencies in all groups converged and dropped dramatically. As in the P18 water maze analysis, stop times (i.e., time spent treading water) during this first trial were significantly lower in ketamine-treated rats [Drug effect: F(1, 62) = 6.645; p = 0.012]. A subsequent examination of the data excluded the time spent treading water in swim-time, swim-distance and swim-speed analyses. On the first trial (see Figure 3), ketamine-treated rats found the platform significantly faster than saline controls [Drug effect: F(1, 67) = 7.28; p = 0.009] and swam shorter distances to do so [Drug effect: F(1, 67) = 8.89; p = 0.004]. Animals injected on E18 were generally quicker to find the platform [Treatment Age effect: F(1, 67) = 10.55; p = 0.002] and swam more direct routes to the platform [Treatment Age effect: F(1, 67) = 5.89; p = 0.018] than were animals injected on E19.

Post-hoc analyses indicated that rats treated with ketamine on E18 reached the hidden platform significantly faster and swam shorter distances than saline-treated controls as well as rats treated with ketamine on E19 (see Figure 3). Rats treated with ketamine (on either E18 or E19) also exhibited significantly fewer failures to reach the hidden platform (within the 90-second limit/trial) than did saline control animals [t(66) = 1.86, p = 0.034 (one-tail test)]: ketamine-treated Mean ± SEM = 0.71 ± 0.05 failures/10 trials; saline-treated Mean ± SEM = 0.24 ± 0.07 failures/10 trials.

The short latencies to mount the platform cannot be attributed to faster swimming speeds. On the first, second and third water maze trials (i.e., the only ones analyzed for swim speeds), P81 rats that were treated with ketamine on E18 did not swim significantly faster than any of the animals in the other treatment groups. For example, the swim speeds for trial 1 were: [E18/ketamine: 34.30 ± 3.59 cm/sec; E18/saline: 37.97 ± 1.85 cm/sec; E19/ketamine: 30.47 ± 2.40 cm/sec; E19/saline: 32.92 ± 2.23 cm/sec (Mean ± SEM)]. Fatigue did not seem to play a role in the group differences since swim speed remained stable in all groups over the first 3 water maze trials on P81.

### Locomotion

A single ketamine treatment during the perinatal period had long-lasting effects on spontaneous locomotor movements. Ketamine's effects depended on the age of behavioral testing as well as the age of the drug treatment. Horizontal movements (i.e., line crossings) were more prominently influenced by perinatal ketamine than were vertical movements (rearing).

#### P11 locomotor tests

**Figure 4:**
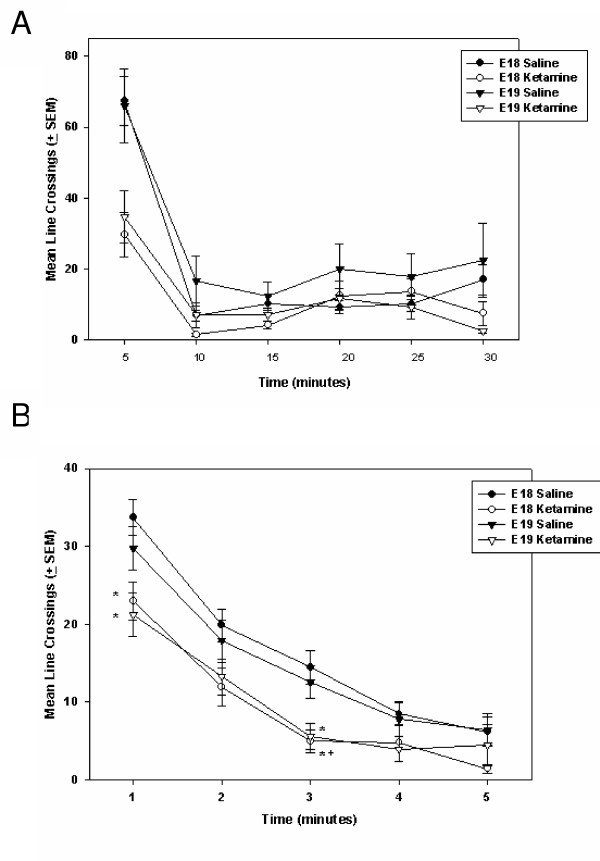
**Spontaneous horizontal locomotor activity of P11 rats treated (through the maternal circulation) with either 100 mg/kg ketamine HCl (i.p.) or saline on either E18 or E19. Panel A **illustrates the entire 30-minute test. In only the initial 5-minute observation period, rats treated with ketamine *in utero *crossed significantly fewer lines than did the saline control animals. After this initial period of habituation, indicators of horizontal movement declined and group scores converged. **Panel B **is a minute-by-minute illustration of the first 5 minutes of locomotor activity. In a time-dependent manner, rats treated with ketamine *in utero *crossed significantly fewer lines than did the control animals. Habituation to the open field is represented by a rapid decline in movement. * = Significantly different from E18/saline group; + = significantly different from E19/saline group. Data were analyzed using a three-way ANOVA [Drug (100 mg/kg ketamine HCl, saline control) X Treatment age (E18, E19) X Time] with time blocks as the repeated time factor and compensation for unequal Ns. If the repeated-measure ANOVA revealed a significant trial effect (indicating a change over time) a two-way ANOVA [Drug (100 mg/kg ketamine HCl, saline control) X Treatment age (E18, E19)] was run to analyze the group differences during a particular trial. Individual group comparisons were accomplished by using the Tukey-Kramer test for Multiple Comparisons. An α = 0.05 was used throughout these analyses. Variance indicators are the Standard Error of the Mean (SEM).

P11 rats treated prenatally with ketamine showed habituation to the open-field test chamber and exhibited reduced horizontal movements overall (see Figure 4). After being placed in the activity chamber, P11 rats decreased their horizontal movements (i.e., line crossings analyzed in 6, 5-minute blocks) significantly over the 30-minute test session [F(5, 405) = 80.66, p < 0.0001]. In fact, locomotor activity of all treatment groups was reduced to very low levels (typically <5 line breaks/min) after the first 5 minutes (Figure 4B).

Ketamine-treated rats were also generally less active than saline-injected controls [F(1, 81) = 7.79, p = 0.007]. However, there was a significant interaction between drug treatment and the block of time in which the locomotor measurement was made [F(5, 405) = 6.26, p = 0.0002]. At the end of 5 minutes, P11 rats reduced their spontaneous locomotion to approximately 20% of its original level. For this reason, we performed a minute-by-minute analysis of the first 5 minutes in the open field apparatus (see Figure 4B). Once again, there was a significant decrease in horizontal movement over the first 5 minutes in the chamber [F(4, 336) = 143.13, p < 0.001]. Generally, ketamine treatment caused a significant decrease in line crossings as compared to saline-injected controls [Drug effect = F(1, 84) = 12.77, p = 0.0006]. A Drug X Time Block interaction [F(4, 336 = 3.21, p = 0.01] revealed that the largest group differences were exhibited within the first 3 minutes (see Figure 4B for individual group comparisons). There was not a significant difference in the spontaneous horizontal locomotor responses of E18- and E19-ketamine treated rats.

#### P60 locomotor tests

**Figure 5:**
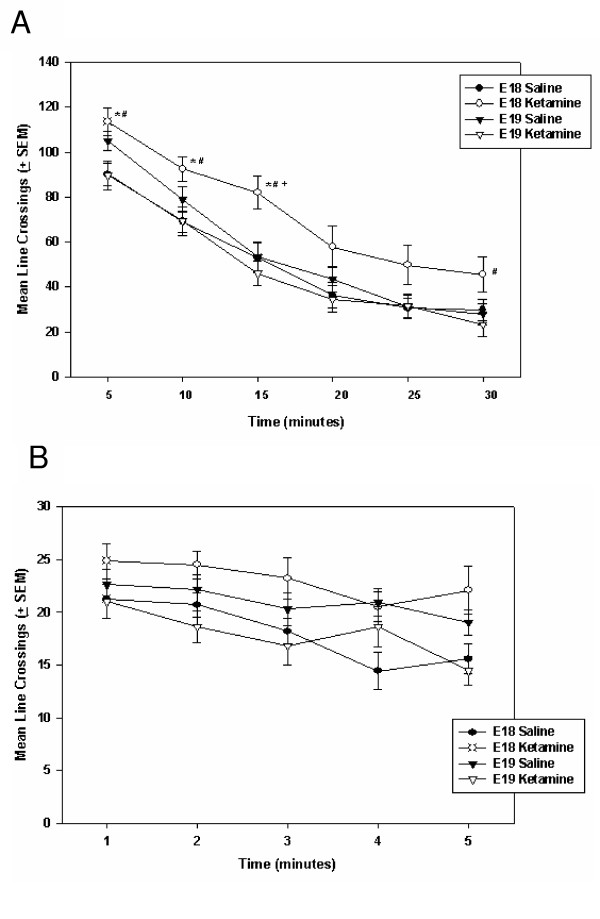
**Horizontal locomotion of P60 rats treated (through the maternal circulation) with either 100 mg/kg ketamine HCl (i.p.) or saline on either E18 or E19. Panel A **illustrates the entire 30-minute test. E18 rats treated with ketamine were significantly more active than were animals in the other treatment groups. These effects were most prominent at particular time periods. * = Significantly different from E18/saline group; # = significantly different from E19/ketamine group. + = significantly different from E19/saline group. **Panel B **is a minute-by-minute illustration of the first 5 minutes of locomotor activity. In all treatment groups, there is a significant decline in locomotion over the first 5 minutes of testing. There is also a significant interaction between drug treatment and subject age at time of treatment – indicating that rats treated with ketamine on E18 are more active than both E18 saline-control rats and E19 rats that received ketamine. Data were analyzed using a three-way ANOVA [Drug (100 mg/kg ketamine HCl, saline control) X Treatment age (E18, E19) X Time] with time blocks as the repeated time factor and compensation for unequal Ns. If the repeated-measure ANOVA revealed a significant trial effect (indicating a change over time) a two-way ANOVA [Drug (100 mg/kg ketamine HCl, saline control) X Treatment age (E18, E19)] was run to analyze the group differences during a particular trial. Individual group comparisons were accomplished by using the Tukey-Kramer test for Multiple Comparisons. An α = 0.05 was used throughout these analyses. Variance indicators are the Standard Error of the Mean (SEM).

Horizontal movements of ketamine-treated rats tested on P60 varied depending on when, during the fetal period, they had received the drug (see Figure 5). As was the case during the P11 tests, horizontal movements decreased significantly over the 30-minute test [F(5, 340) = 163.12, p < 0.0001]. There was both an overall effect of subject age at time of drug injection [F(1, 68) = 4.37, p = 0.04] and a Treatment Age X Drug interaction [F(1, 68) = 10.37, p = 0.002]. Over the course of this 30-minute test (Figure 5A), E18 fetuses treated with ketamine were generally more active in their horizontal movements than were animals treated with saline on this day of embryonic development. Further, rats exposed to ketamine on E18 exhibited significantly more horizontal movement than did E19 rats treated with either saline or ketamine. Rats injected with saline on E18 or E19 did not exhibit significant differences in line crossings when tested on P60.

An analysis that focused on the first 5-minutes of this P60 locomotor test (Figure 5B) revealed a significant interaction between drug treatment and subject age at time of treatment [F(1, 70) = 14.13, p < 0.001]. Multiple comparisons revealed that rats treated with ketamine on E18 were more active than both E18 saline-control rats and E19 rats that received ketamine. These data reveal a very different pattern of horizontal locomotor responses exhibited by ketamine-treated rats depending on the day during fetal development that they received the drug. There were no significant group differences in rearing movements on P60.

## Discussion

The data presented here suggest several age-dependent effects of early ketamine treatment – effects that depend on not only the day of behavioral testing but also the day in embryonic development when the NMDA receptor antagonist was administered. During the initial stage of the second water maze test (on P81), rats treated with ketamine on E18 found the hidden platform more quickly than did animals receiving the same treatment on E19. Moreover, they exhibited enhanced maze performance compared to both groups of saline-treated rats. It is important to note that ketamine treatment on E18 did not induce faster swim speeds. Rather, the animals swam more direct routes to the hidden platform.

Effects of ketamine on spontaneous open-field locomotion were also age-dependent. In neonatal animals (P11), ketamine administration *in utero *reduced subsequent spontaneous movement. This effect was subtle (i.e., only in evidence within the first 3-minutes of testing) and did not depend on the subject's age at the time of the drug's administration. However, when these animals were re-tested on P60, the rats that received ketamine on E18 both moved more than the rats that received ketamine on E19 and also moved more than saline-injected controls.

First, these data reveal long-term behavioral effects of a single dose of ketamine administered *in utero*. Drug-induced effects on water maze learning were observed over 11 weeks after birth and locomotor effects were documented 9 weeks *post partum*. These findings are consistent with others indicating that early NMDA receptor blockade may produce behavioral alterations that are detectable in adulthood [23,25,26]. Second, these data are consistent with the hypothesis that the behavioral effects of NMDA receptor blockade depend on the day in embryonic development when the antagonist is administered. In particular, some of the findings reported here extend our previous work indicating that ketamine treatment on E18 may have different effects than administration of the drug on E19 [21]. The same dose of ketamine administered in the current study potentiated conditioned motor responses of neonates if the drug had been given (through the maternal circulation) on E18. However, ketamine impaired acquisition of this learned response if it was administered one day later on E19 [21]. Similar age-dependent effects have been reported using different behavioral indicators of learning [22,27].

The effects of early ketamine treatment on locomotion are apparently not consistent throughout postnatal development. Ketamine reduced locomotor movements in P11 rats but later (P60) selectively enhanced locomotion of animals that received the drug on E18. The reasons for this change in responding are unclear. In order to accommodate the different size of the animals at P11 and P60, there were differences in the dimensions of the open field chambers used at each test. Also, the chamber walls were clear during the P11 test and opaque at P60. But beyond these differences in apparatus, maturation clearly brings with it a variety of capabilities and propensities many of which can modulate motor responding. For example, at the end of 5 minutes, P11 rats reduce their spontaneous locomotion to approximately 20% of its original level. However, P60 rats are 80% as active during this same time period. These data indicate a general tendency for young rats to habituate (or fatigue) more rapidly than older rats. Other behavioral studies have revealed toxin-induced performance impairments that reveal themselves only at certain stages of early postnatal development but not at older ages [28]. More recently, Beninger et al. [29] reported that rats administered MK-801 on P3 and tested pre- (P35) and post-pubertally (P56) exhibited different locomotor responses to amphetamine depending on the time of the behavioral test.

Our measures of swim speed may offer some insights regarding the relative motor capacities and motivation of our animals. Swim speeds did not significantly differ between animals previously treated with ketamine or saline. Likewise, fetal age at the time of the drug treatment did not influence speed of swimming. Instead, rats reduced their latencies to mount the platform by swimming more-direct routes. Thus, the water maze data reported here are less likely a reflection of the animal's capacity or motivation to get to the platform and more likely a reflection of learning ability.

It should be noted that water maze performance may be influenced by a number of factors beyond cognitive ability. For example, drug-induced alteration of visual acuity, motivation or motor capacities can alter performance of this task [30]. The literature suggests that early NMDA receptor blockade may alter development of the visual system [31,32]. But measures of actual visual acuity following a single occurrence of NMDA receptor blockade in the developing brain are lacking. The available data suggest that visual plasticity is more significantly altered by NMDA receptor antagonism than are visual maps [33] or neural activity per se [34]. Our water maze procedures did not necessarily place demands on the rat's visual system. The location of the hidden platform was not changed from trial to trial or between the P18 and P81 tests. Therefore, once the platform was located, our subjects could have adopted motor strategies to reach the goal on subsequent trials. Although water maze performance is typically cited as an indicator of spatial learning, our paradigm does not eliminate the possibility that other types of learning may also be involved.

We used accepted statistical methods to avoid spurious inflation of sample size and to control for litter effects [35,36] (see Methods section below). However, due to constraints of resources, the small number of litters employed may have reduced our power to detect subtle differences in performance. Thus, this report should be viewed as a conservative account of drug- and age-influenced changes in behavior.

It is worth noting that it was initial responding on the water maze and in the locomotor test chamber that was most sensitive to our fetal ketamine treatment. ketamine-treated rats exhibited significantly faster times to reach the hidden platform (at age P81) – but only on the first trial. Likewise, P11 rats treated with ketamine as fetuses, exhibited fewer locomotor movements than did saline controls – but only during the first 5 minutes of our test. Our laboratory [37], as well as other investigators [38] have reported a role for NMDA receptors in the determination of novelty. The current data seem to suggest that these effects may extend to various behavioral testing paradigms. Moreover, since our tests were conducted weeks after fetal ketamine treatment, our data indicate the persistence of ketamine's effects on initial responding.

What neural, or other, mechanisms might subserve the behavioral phenomenon outlined here? NMDA receptor populations and physiology are neither static nor mature during the perinatal period and blockade of these receptors during particular days of development may produce quite different effects [39,40]. For example, Sircar [41] has shown that the binding of [^3^H] MK-801 (a potent/selective NMDA receptor antagonist) in synaptosomal membranes is differentially altered by glutamate (and other) agonists during various periods of development. These data build on previous findings [42] indicating a dramatic change in the number of PCP-binding sites in fetal rat brain between the ages of E18 and E19. This is the same time frame in which ketamine's effects on memory change so significantly. Could these developmentally linked changes in NMDA receptor populations and functional roles mediate the ketamine paradox as well as the behavioral phenomena presented here?

The identification of several NMDA receptor subtypes with different functional roles and different patterns of expression during the perinatal period may also eventually reveal aspects of the ketamine paradox's physiological substrate [43-54]. Could a drug-induced change in the population and/or distribution of NMDA receptor subtypes mediate the ketamine paradox as well as long-term behavioral effects? The current data do not address this point directly. However, NR2B NMDA receptors (which are known to be involved in learning, in general, and taste memory formation, in particular) [55-57] have been identified as being especially sensitive to upregulation following pharmacological antagonism [58,59]. Further, other laboratories have reported that NMDA exposure can produce a reduction in NMDA receptors within 4 hours of exposure [60]. Our data suggest the potential usefulness of studies aimed at correlating ketamine-induced changes in NMDA receptor subtype populations with behavioral outcomes recorded at several times in development. Such experiments are currently underway in our laboratory.

It should also be noted that ketamine can influence maternal and fetal physiology in ways that go beyond the drug's well-known effects on NMDA glutamate receptors [1]. Pulmonary vasodilator responses have been recorded following ketamine administration [61]. The drug can also alter uterine tone in pregnant ewes by increasing cardiac output and mean arterial pressure [62]. Although these cardiovascular effects were slight and transient, they may have contributed in yet-unknown ways to some of the long-term behavioral changes we report here. Likewise, ketamine not only affects NMDA receptors but may also inhibit non-NMDA glutamate receptors [63], the high-affinity states of the dopamine D2 receptor, and other G-protein-linked receptors [64]. While ketamine's actions on NMDA receptors are certainly prominent, acute changes in vascular tone and the drug's actions on other brain receptors are capable of influencing fetal development in ways not addressed by the current experiments.

If early ketamine exposure influences NMDA receptor populations or functioning, post-synaptic second messenger pathways would also be engaged as mediators of behavioral change [65]. Downstream calcium and calmodulin signaling, calcium-dependent kinases, and ultimately changes in gene expression are known to produce synaptic restructuring [66]. This cascade of NMDA-receptor-initiated biochemical events provides a likely avenue for further investigation as we examine the physiological substrate of the behavioral phenomena described here.

The variables of subject age and ketamine dose interact in complex ways to produce predictions of neurotoxicity. If NMDA antagonists are used to suppress neuronal activity during a critical developmental period of synaptogenesis, the timing and sequence of synaptic connection is disrupted [67]. This causes neurons to receive an internal signal to commit suicide – a form of programmed cell death called apoptosis [68]. Ketamine, and other NMDA receptor blocking drugs, are reported to produce these neurotoxic effects [69] under certain circumstances. In the rat, the period of brain sensitivity is largely confined to the postnatal period (i.e., from P1 to P14) [70]. Our single dose of ketamine was administered on either E18 or E19, i.e., subject ages that, to the best of our knowledge, have not been systematically manipulated in studies aimed at investigating ketamine's ability to produce apoptosis. These studies should be done to confirm the role that apoptosis may/may not play in producing the long-term behavioral changes we report here.

In addition to subject age, the dose of ketamine is another important factor in determining the likelihood of apoptosis induction as well as the generalizability of our data to clinical settings. In order to produce an increase in apoptotic neurons in neonatal rats, ketamine must be administered in multiple injections over a period of 9 hours [13]. Our study used a single dose of ketamine (delivered to a pregnant rat) that was significantly higher (100 mg/kg) that those used previously in neonates [69]. However, from previous biochemical studies we know that our dosing regimen in pregnant rats [27] produced a concentration of fetal brain ketamine roughly comparable to that seen in blood following repeated doses of 20 mg/kg administered to neonatal rats (14 μg/g) [69]. These blood levels were approximately seven-fold greater than anesthetic blood levels in humans [71,72]. Therefore, by extrapolation, we may predict that our dose of ketamine produced tissue levels of the drug that significantly exceeded those typically produced in human patients who encounter the drug in a clinical setting. Of course, this does not eliminate the possibility that the human recreational use of ketamine (street name: "Special K") [68] may produce blood and brain levels that are significantly higher than those encountered in the clinic. Nor does it exclude the possibility of differing drug sensitivities of rats and humans. Both these factors will influence the clinical relevance of the studies reported here.

## Conclusions

These studies were aimed at determining the long-term behavioral effects of ketamine administration on E18 and E19 as a means of assessing the durability, intensity and generalizability of the ketamine paradox [21]. Our previous work indicated that ketamine administration enhanced the formation of a conditioned taste aversion in E18 fetuses but not those treated on E19 or later [20-22,29]. The current data reveal several subtle, but consistent, residual behavioral changes produced by of a single large dose of ketamine administered during the rat's late pre-natal period. In terms of ketamine effects on spontaneous locomotion, we found that, irrespective of the day of fetal dosing employed, ketamine reduced horizontal movements when animals were tested on P11. However, when the animals were tested later, on P60, rats that had received ketamine on E18 differentiated themselves from the E19 ketamine-treated animals (and saline-treated controls) by exhibiting an increase in locomotion – especially in the early minutes of behavioral testing in the open field. Ketamine's long-term influence on water maze learning/retention was limited in scope, but palpable, on the first trial of the P81 test.

Despite the reliable enhancement of CTA learning that has been reported in fetuses and neonates treated with ketamine on E18 [29], this same dosing regimen produced limited improvements in learning/retention of a water maze when the animals were tested as young adults. The usefulness of ketamine as a cognitive enhancer, administered in the perinatal period, appears to be limited not only by its known toxic effects at critical stages of development [68] but also by its influence on spontaneous movement and the drug's weak memory-enhancing properties over the long term.

## Methods

### Subjects

The subjects were Sprague-Dawley rats (male and female) obtained from timed pregnant female rats supplied by Zivic Laboratories (Zelienople, PA). Litters (N = 2/treatment group) were not culled and ranged in size from 9 to 13 pups. [See behavioral testing sections below for details about the number of animals in each treatment group.] The variable number of subjects/group was due to different litter sizes and several logistical constraints that did not always allow the testing of all rats in each litter. Statistical adjustments were made in order to compensate for the unequal Ns in the treatment groups (see below). The date of conception (i.e., the date a vaginal plug was first detected) was designated as "embryonic day 0" (E0). Postnatal day 0 (P0) was the day of birth (typically E21.5). The pregnant animals from which our subjects were derived were individually housed in plastic 'shoe-box' cages (44.45 cm long × 21.59 cm wide × 20.32 cm high). After birth, perinatal rats were housed with the dam until they were sexed and weaned between P21-25 (this is within recommended weaning dates, see [73], at which point the pups were group-housed (separated by sex) in the standard-sized cages described above. Throughout the experiment home cage temperature was maintained at 23–26°C under a 12:12-h light:dark cycle (lights on at 0600 h).

The Baldwin-Wallace College Institutional Animal Care and Use Committee approved these experiments. The animals involved in this study were procured, maintained and used in accordance with the Animal Welfare Act and the *Guide for the Care and Use of Laboratory Animals*, prepared by the Institute of Laboratory Animal Resources – National Research Council.

### Drug treatments

Pregnant dams received ketamine HCl (100 mg/kg, i.p.; Sigma Chemical Company), or an equal volume of physiological saline, (0.9% NaCl, i.p.) on either E18 or E19. Saline injections controlled for the stress of pre-natal manipulation. Thus, there were 4 treatment groups designated hereafter as follows: E18/ketamine, E18/saline, E19/ketamine, or E19/saline. Rats from two litters were used in each of the treatment groups. In order to separate out treatment effects from litter effects special statistical measures were employed (see *Statistical Analyses*, below) [35,36]. The dose of ketamine employed (100 mg/kg, i.p.) was selected based on previous experiments [21] in which the drug produced very different behavioral effects when administered to E18 or E19 fetuses through the maternal circulation. Using high-pressure liquid chromatography (HPLC) measures we have previously determined the level of ketamine (approximately 14 μg/g tissue) found in the brains of fetuses following a maternal injection of 100 mg/kg ketamine during the late pre-natal period [27].

### Behavioral testing

We recorded performance in a water maze and also spontaneous locomotor movements in an open field. Each of these behavioral tests was conducted twice, i.e., once in each of two different time periods: *Juvenile Period *(pre-pubertal), within the first three weeks after birth and *Young-adult Period *(post-pubertal), between 2–3 months of age. We selected these two behavioral test periods based on knowledge of the patterns of development of NMDA receptor subtypes. The NR2B receptor subtype has been implicated in learning and memory [55]. These receptors are present during the late pre-natal period and they rise steadily up to P20 when they achieve adult levels [43,74]. Therefore, our behavioral measures sampled times both before and after maturation of this receptor system. The behavioral tests conducted within the juvenile or post-pubertal periods were separated in time (by a minimum of 1 week) to reduce the influence of one on the other.

Most, but not all, animals were tested and then retested. However, statistical analyses indicated that there were no differences between the animals that were tested once or twice. Therefore, these groups were combined for subsequent statistical analyses. Additional behavioral testing (i.e., conditioned taste aversion) was performed on some of these animals but there were some logistical problems with the experiment. These data did not reveal reliable group differences and are not reported here.

#### Water maze

At age P18, and then again at P81, we evaluated water maze performance by testing the following number of rats per group: *P18*: E18/ketamine: (N = 12); E18/saline: (N = 16); E19/ketamine: (N = 14); E19/saline: (N = 15); *P81*: E18/ketamine: (N = 12); E18/saline: (N = 22); E19/ketamine: (N = 17); E19/saline: (N = 20). Our data corroborate other studies indicating that rats younger than P20 are capable of learning a water maze [75,76].

The water maze was an oval stock-watering tank (manufactured by Rubbermaid, Inc.) measuring 94 cm × 74 cm × 60 cm deep. It was sized to shorten swim distances to the hidden platform and reduce the likelihood of fatigue in our young animals. The tank was filled to 39.5 cm (1 cm above a hidden platform) with water that was made opaque and white by adding 710 ml of evaporated milk (Nestle's Carnation^® ^brand). The water temperature was maintained at 26 ± 1°C. The escape platform was a clear plastic disc (12.5 cm diameter × 1.2 cm) mounted on a stand. The platform remained in the same location (approximately 10 cm from the side of the tank) throughout the test session. The edges of the platform had white rubberized tape attached in order to aid the rats as they mounted it. The tank was in a room lighted with fluorescent lights and surrounded by a rich array of laboratory furnishings.

All test sessions were video recorded and the tapes were later used for analysis of latency to escape, stop time, time on platform, path length and swim speed (see *Statistical Analyses *below). "Escape latency" was defined as the time (in seconds) it took the rat, once in the water, to mount and gain balance on the platform. "Stop time" was the total time/trial that subjects spent treading water (i.e., not making forward progress). "Time on the platform" was defined as the time (up to 30 sec) the animal remained on the platform after initial mounting. "Path length" was the total distance (cm) swum before the subject mounted the platform. "Swim speed" was expressed in cm/sec and reflected the rate of forward progress towards the platform.

At the beginning of each water maze test session, a rat was placed in the water facing the wall of the tank opposite the one near the hidden platform. A swim trial lasted until the rat reached the hidden platform or until 90 seconds had passed. If the animal reached the platform in the allotted 90 seconds, it was allowed to remain on the platform for up to 30 seconds and was then returned to a holding cage (a dry, plastic "shoe-box" cage). The holding cage sat upon a heating pad set at 33.5°C., producing a floor temperature of approximately 28.5 ± 1°C. If the animal did not reach the platform in 90 seconds it was removed from the water and returned to the cage. If the animal jumped off the platform before 30 seconds, it was removed from the water and returned to its holding cage. All rats were given a 60-second rest period before the next trial was initiated. Each rat experienced ten swim trials during each of the two test sessions. At the end of the 10 trials, each rat was thoroughly dried with a towel and a blow dryer and then returned to its home cage. Escape latencies and time on the hidden platform were recorded for each trial.

#### Spontaneous locomotion

At age P11, we measured the spontaneous locomotor activity of the following number of rat pups in each group: E18/ketamine: (N = 18); E18/saline: (N = 25); E19/ketamine: (N = 21); E19/saline: (N = 24). The test chamber consisted of a plastic 'shoe-box' cage (44.45 cm long × 21.59 cm wide × 20.32 cm high) with transparent walls and open top. This chamber had a grid on its floor composed of 3 × 6 squares (each measuring 7.1 cm × 7.1 cm). Young rats have limited abilities to thermoregulate [77]. Therefore, the activity chamber was placed on a heating pad set at 33.5°C., producing a floor temperature of approximately 28.5 ± 1°C. An individual pup was initially positioned in the center square of the chamber. Locomotor activity was recorded for 30 minutes. Test sessions were video recorded and tapes were later scored (see below). After each session, the animal was weighed and then returned to its home cage. In preparation for the next animal, the activity chamber was cleaned by spraying the cage with 50% ETOH, wiping it clean with paper towels, and allowing it to air-dry for approximately 10 minutes.

At age P60, we again measured the spontaneous locomotor activity of the following number of rats in each group: E18/ketamine: (N = 12); E18/saline: (N = 21); E19/ketamine: (N = 17); E19/saline: (N = 22). For this second test, a larger test chamber (64 cm long × 46 cm wide × 42 cm high) was used. The walls were opaque plastic and the top open. This chamber had a grid on its floor that consisted of 3 × 3 rectangles (each measuring 21 cm × 15 cm). As before, rats were individually placed in the center square of the chamber at the beginning of the 30-minute test session. After each session, the animal was weighed and then returned to its home cage. In preparation for the next animal, the activity chamber was cleaned as described above.

Videotapes of locomotor movements were later viewed and independently scored by observers blind to the experimental condition of the animal. We counted line crossings to assess the amount of horizontal locomotion exhibited by each animal. A "line-cross" was counted when any part of the rat, except the tail, crossed a line. Rearing was operationally defined as any time the rat raised both front paws from the chamber floor. In P11 rats, rearing was very rare and therefore not scored. However, this behavior was recorded during the P60 test.

### Statistical analyses

Escape latencies and time on the hidden platform were recorded for each trial in the water maze. Group differences in water maze performance were most evident early in training. After the first few trials, rats in all treatment groups moved promptly to the hidden platform with a latency of less than 20 seconds. For this reason, our statistical analyses focused on the initial trials of each session. Animals removed from the maze after not finding the hidden platform in 90 seconds were, nevertheless, assigned a time of 90 seconds for purposes of data analysis.

Swim distances, swim speeds, and time spent treading water (stop times) were also calculated for the first 3 swim trials. Towards this end, videotapes were viewed and independently evaluated by raters blind to the experimental condition of the animal. Swim paths were hand-drawn on acetate placed on a video screen during playback of the videotape. The paths were digitized using a light pen providing input to NIH Image software (Bethesda, MD). The lengths of the paths were compared with a calibrated length on the video record in order to calculate the swim distance. An evaluation of the inter-rater reliability of our video scoring methods indicated a high correlation [r(10) = 0.924, p < 0.001]. These methods produced swim distances that were not significantly different [t(9) = 1.02, p > 0.05] between observers. For some of our swim speed analyses, we subtracted out any time that the animal stopped swimming mid-trial, and treaded water at the side of the tank. Dividing the swim distance by the adjusted time to mount the hidden platform produced swim speed.

Two observers, blind to the experimental condition of the animals, evaluated each of the tapes of animals locomoting in the open field by counting line crossings and rears. The counts of these 2 observers were then averaged and this data point was used in our statistical analysis. There was high degree of correlation between the ratings of our 2 observers. P11 locomotor test: r(85) = 0.91, p < 0.01; P60 locomotor test: r(72) = 0.90, p < 0.01.

Unless otherwise stated, locomotor data for the two different test periods (P11 and P60) and water maze data for the 2 different test periods (P18 and P81) were analyzed via separate repeated-measures, three-way ANOVAs [Drug (100 mg/kg ketamine HCl, saline control) X Treatment age (E18, E19) X Time] with time blocks as the repeated factor and compensation for unequal Ns.

We used several rats from each litter and employed statistical corrections in order to avoid spurious inflation of sample size [35]. Since all the rats in a particular litter received the same drug treatment, we included litter as an independent and nested factor in the analysis. This approach controls for litter effects and offers a direct statistical test of the significance of such effects [35]. Denenberg [36] has recommended this procedure to allow the partitioning of litter and treatment effects and thereby allowing investigators to make use of the data from multiple animals in a litter. When significant litter effects were detected, we used the Mean Square (MS) associated with the litters as the error term rather than the MS of the subjects. However, if there was not a statistically significant litter effect, the data were subsequently reanalyzed without this component as part of the general linear model (GLM; software provided by SAS™, SAS Institute, Carey, NC; and, SPSS™ Inc., Chicago, IL).

An initial inclusion of subject sex as a factor in our statistical analyses indicated no significant differences between male and female subjects. Therefore, the subsequent analyses reported here were run without this factor.

Platform navigation for juvenile rats might be more challenging than the task presented to older rats. A small water maze was used in these studies and the same start-to-platform distance was used for all tests. Still, the P18 rat may have found it significantly more challenging than the P81 rat to traverse this distance. For this reason, we intentionally avoided comparing the water maze data of our juvenile and young-adult rats. Likewise, we did not make statistical comparisons between the locomotor responses of animals run at the 2 different ages since the use of different-sized apparatuses would presumably influence these data.

If a repeated-measure ANOVA revealed a significant trial effect (indicating a change over time) a two-way ANOVA [Drug (100 mg/kg ketamine HCl, saline control) X Treatment age (E18, E19)] was run to analyze the group differences during a particular trial. Individual group comparisons were accomplished by using either the Tukey-Kramer Multiple Comparisons Test or t-tests [78] using the Bonferroni compensation for multiple comparisons. Our previous studies with ketamine-treated fetuses lead us to *a priori *hypotheses regarding possible behavioral differences between rats treated with ketamine on E18 versus E19 [21]. When *a priori *planned comparisons were made, one-tail probabilities were computed. An α = 0.05 was used throughout these analyses presented here.

## Authors' contributions

GAM designed the studies, performed some of the behavioral work, conceptualized the statistical analyses and drafted the manuscript. CK helped design the studies, performed most of the behavioral work, assisted with statistical analyses and the drafting of the manuscript. CM supervised most of the behavioral work, assisted with statistical analyses and the drafting of the manuscript. AS supervised and performed much of the behavioral work. AY performed most of the behavioral work. DL-F assisted with the statistical analyses. EV assisted with the behavioral work. BW assisted with the behavioral work. JMB performed aspects of the behavioral work and assisted with the drafting of the manuscript.
